# Detection of Measurable Residual Disease Biomarkers in Extracellular Vesicles from Liquid Biopsies of Multiple Myeloma Patients—A Proof of Concept

**DOI:** 10.3390/ijms232213686

**Published:** 2022-11-08

**Authors:** Rui Bergantim, Sara Peixoto da Silva, Bárbara Polónia, Mélanie A. G. Barbosa, André Albergaria, Jorge Lima, Hugo R. Caires, José E. Guimarães, M. Helena Vasconcelos

**Affiliations:** 1 i3S—Instituto de Investigação e Inovação em Saúde, University of Porto, 4200-135 Porto, Portugal; 2 Cancer Drug Resistance Group, IPATIMUP—Institute of Molecular Pathology and Immunology of the University of Porto, 4200-135 Porto, Portugal; 3 Clinical Hematology, Hospital Center of São João, 4200-319 Porto, Portugal; 4 Clinical Hematology, FMUP—Faculty of Medicine of the University of Porto, 4200-319 Porto, Portugal; 5 Research Innovation Unit, Translational Research & Industry Partnerships Office, i3S—Instituto de Investigação e Inovação em Saúde, University of Porto, 4200-135 Porto, Portugal; 6 Instituto Universitário de Ciências da Saúde, Cooperativa de Ensino Superior Politécnico e Universitário IUCSESPU, 4585-116 Gandra-Paredes, Portugal; 7 Department of Biological Sciences, FFUP—Faculty of Pharmacy of the University of Porto, 4050-313 Porto, Portugal

**Keywords:** multiple myeloma, extracellular vesicles, measurable residual disease, liquid biopsy

## Abstract

Monitoring measurable residual disease (MRD) is crucial to assess treatment response in Multiple Myeloma (MM). Detection of MRD in peripheral blood (PB) by exploring Extracellular Vesicles (EVs), and their cargo, would allow frequent and minimally invasive monitoring of MM. This work aims to detect biomarkers of MRD in EVs isolated from MM patient samples at diagnosis and remission and compare the MRD-associated content between BM and PB EVs. EVs were isolated by size-exclusion chromatography, concentrated by ultrafiltration, and characterized according to their size and concentration, morphology, protein concentration, and the presence of EV-associated protein markers. EVs from healthy blood donors were used as controls. It was possible to isolate EVs from PB and BM carrying MM markers. Diagnostic samples had different levels of MM markers between PB and BM paired samples, but no differences between PB and BM were found at remission. EVs concentration was lower in the PB of healthy controls than of patients, and MM markers were mostly not detected in EVs from controls. This study pinpoints the potential of PB EVs from MM remission patients as a source of MM biomarkers and as a non-invasive approach for monitoring MRD.

## 1. Introduction

Treatment of Multiple Myeloma (MM) has improved substantially, demonstrated by the current sequential and integrative approach using drugs of different classes in combination, which has clear benefits for patients [1]. In recent years, several drugs, such as proteasome inhibitors, immunomodulators, and monoclonal antibodies, have been approved for the treatment of MM, and several others with novel mechanisms of action are currently under pre-clinical and clinical trials, namely bi-specific monoclonal antibodies and chimeric antigen receptor (CAR)-T cells. Despite the unprecedented response rates and prolonged survival achieved with these significant advances in treatment, MM remains incurable and, eventually, all patients inevitably relapse [2].

A deep and sustained complete response (CR) to treatment is the ultimate objective in any phase of MM treatment. Nonetheless, achieving CR is not enough to ensure longer survival in MM. Indeed, the persistence of very low levels of plasma cells (PCs) in patients’ bone marrow (BM), as measurable residual disease (MRD) after treatment, will constitute the soil for a subsequent relapse and are a major cause of drug resistance. Quantifying MRD allows for the assessment of chemotherapy efficacy and clinical outcome, the identification of patients at high-risk of recurrence, and provides important information for therapy decisions [3,4]. MRD evaluation is most important to determine when the myeloma clone is disappearing during treatment (remission) and when the myeloma burden, even low, is reappearing after treatment (relapse) [5,6]. Thus, its routine monitoring has the potential to guide therapeutic decisions [4,7,8].

Indeed, MRD constitutes the most significant predictor of clinical outcome [9,10]. In large meta-analyses and several multivariate analyses in clinical trials, achieving negative MRD is the major and the strongest independent prognostic factor, outweighing classical favorable prognostic factors [9,11,12,13]. MRD is associated with unprecedentedly improved progression free-survival (PFS) and overall survival (OS), regardless of the depth of the International Myeloma Working Group (IMWG) criteria response at the time of MRD evaluation (CR versus above or very good partial response) [14,15], the cytogenetic risk (high or standard risk) [16,17], the time of MRD assessment (at induction, transplant, consolidation, before or after maintenance treatment initiation) [18,19], the status of the MM disease (newly diagnosed or relapsed/refractory disease) [20], or the fact that patients are eligible or non-eligible for transplant [10,19]. Moreover, the real clinical impact of MRD is reproducible in different centers enrolling patients in clinical trials, and also in the clinical practice [21]. 

MRD evaluation can be divided into two approaches: measuring intramedullary disease by multiparameter flow cytometry (MFC) immunophenotyping or by high-throughput/next-generation sequencing (NGS) molecular assessment of immunoglobulin gene rearrangements; or by quantifying extramedullary disease (EMD) using functional imaging methods [21,22,23]. Using MFC makes it possible to identify myelomatous PCs based on light-chain clonality of phenotypically aberrant tumor cells with a sensitivity of 10^−6^ using next-generation flow (NGF) cytometry [24,25,26]. Clonal immunoglobulin gene rearrangements, initially identified by the allele-specific oligonucleotide polymerase chain reaction (AS-PCR), are now detected by NGS that performs millions of reads of DNA fragments with a sensitivity of 10^−6^. Both NGF and NGS lack standardization over different laboratories but yield similar results [23,27,28]. The 18-fluoro-2-deoxyglucose positron emission tomography/computed tomography (FDG-PET/CT) is the optimal method to evaluate the disease outside the BM, allowing for lesions with metabolically active disease to be distinguished, which is mostly useful for patients with EMD; nonetheless, this approach has not yet been validated in randomized clinical trials and cannot be currently used to guide therapeutic decisions [29,30]. MRD determination by either NGF or NGS relies on BM samples and specific pitfalls can compromise its overall success [21]. MM is characterized by a patchy pattern of BM infiltration and different clonal PCs may reside in different areas of the BM, reflecting its spatial molecular heterogeneity [31]. When using NGF and NGS, no EMD is assessed, which may hamper an MRD result from being truly negative, mainly in relapses. Moreover, BM can be hemodiluted and, to minimize false-negative MRD results, the result should be confirmed in a second assessment [21]. These limitations allied to the painful and discomforting collection of BM samples led to efforts to identify alternative approaches using peripheral blood (PB).

Extracellular Vesicles (EVs) are a group of lipid bi-layer circular particles with different biogenesis that are heterogenous in size and content. EVs include exosomes (with endosomal origin and sizes ranging from 30 to 100 nm), microvesicles (with plasma membrane origin and sizes ranging from 50 to 2000 nm), or even apoptotic bodies (with both cytosolic components and nuclear fragments and sizes ranging from 50 to 5000 nm) [32,33,34]. Initially, EVs were considered as cellular waste with no biological activity, but now are recognized as essential players in the intercellular communication mediated by their cargo [35,36,37,38]. In fact, EVs are responsible for the horizontal transfer of phenotypes between cells since they transport cellular contents from the donor cells (e.g., proteins, microRNAs, or fragments of DNA), which may be incorporated by recipient cells [39,40]. EVs are released by all cell types, both normal and malignant, and have recently emerged as a possible source of blood biomarkers for several diseases, particularly cancer [32,41,42]. In fact, cancer cells produce and release a higher amount of EVs when compared to healthy control cells [43,44,45,46]. In recent years, several studies have shown how EVs are relevant for several hallmarks of cancer, such as cellular proliferation, immune modulation, or metastasis [35,47,48,49,50]. 

In MM, EVs play a crucial role in mediating the mutual crosstalk between the MM PCs and cells from the tumor microenvironment by transferring active molecules such as lipids, proteins, and regulatory RNAs, thus contributing to MM pathobiology [44,51]. Another example of the relevance of this communication is the interplay between BM mesenchymal stem cells (MSCs) and PCs, in which MM-MSCs-derived EVs are taken up by MM-PCs, causing proliferation, survival, and migration of the latter by activating oncogenic factors [52,53]. EVs secreted by MM-PCs also contribute to angiogenesis by transferring high amounts of vascular endothelial growth factor (VEGF) to endothelial cells [54]. Interestingly, drug-resistant cells appear to release higher amounts of EVs than drug-sensitive ones [55,56]. Moreover, some studies have shown the transfer of EVs from drug-resistant PCs to sensitive cells, protecting the recipient cells from apoptosis, influencing the activation of several survival pathways, and promoting drug resistance [55].

Considering the flow of communication between MM-EVs and other cells in the BM microenvironment, as well as the dynamic quantity of EVs in relation to disease severity, stage, or treatment phase [57,58,59,60], it is conceivable that MM-EVs and their cargo could be used as potential biomarkers of the disease and to monitor MRD in PB. This study aimed to implement a protocol for the isolation of EVs from the BM and PB of MM patients at distinct disease stages (diagnosis and remission) and to compare the levels of some MRD-associated protein markers between BM and PB EV samples.

## 2. Results and Discussion

### 2.1. EVs Were Isolated from the PB and BM of MM Patients

EVs from the PB and BM of MM patients and EVs from the PB of healthy controls were isolated by Size Exclusion Chromatography (SEC) followed by ultrafiltration (UF) and were characterized by Nanoparticle Tracking Analysis (NTA), Transmission Electron Microscopy (TEM), and Western Blot (WB) in terms of size distribution, morphology, and the presence of well-known protein EV markers to confirm the identity and purity of the isolated EVs.

**Figure 1:**
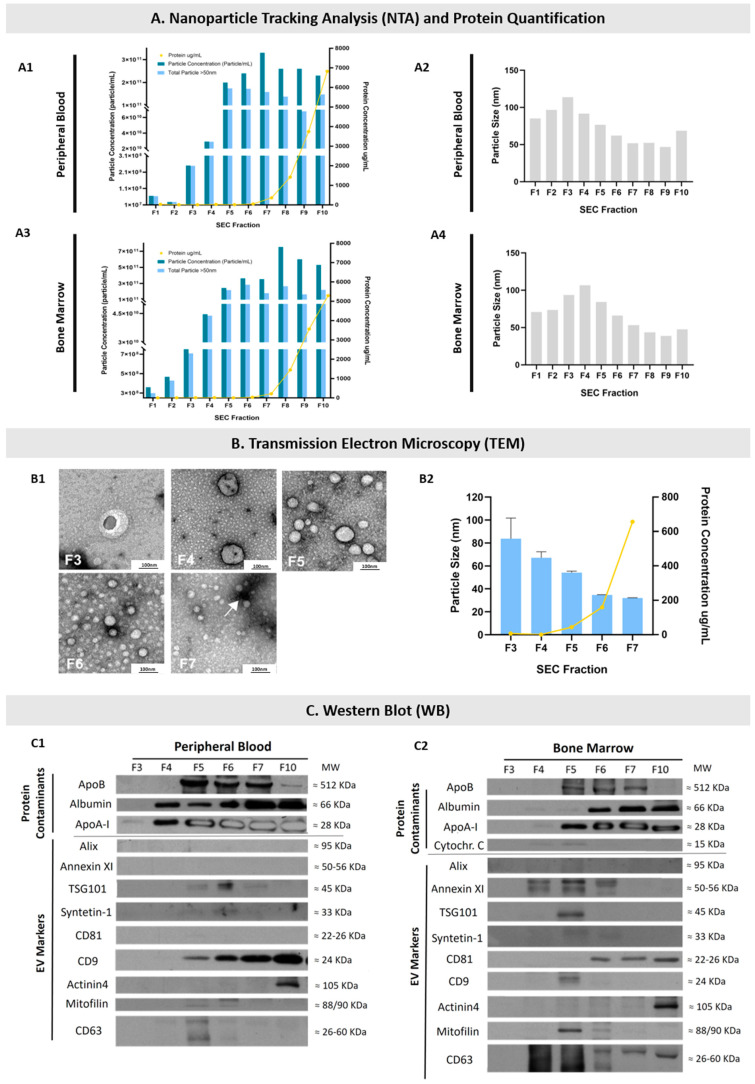
Characterization of the EVs isolated from peripheral blood (PB) and bone marrow (BM) samples by Size Exclusion Chromatography (SEC) and concentrated by ultrafiltration (UF). (**A**) NTA and protein quantification results suggest the presence of EVs from SEC fractions 3 to 6. Graphics on the left show particle concentrations in each SEC fraction (dark blue bars; left axis), the particle concentrations above 50 nm of size (light blue bars; left axis), and the total proteins quantified (yellow dots and lines; right axis) throughout the SEC fractions from a MM patient PB (**A1**) and BM (**A3**) sample. Graphics on the right show the mean particle size distribution in each of the PB (**A2**) and BM (**A4**) eluted SEC fractions analyzed by NTA. (**B**) TEM results confirm the presence of EVs on SEC fractions 3 to 7 isolated from PB samples. (**B1**) Representative images of EVs from SEC fractions 3 to 7 obtained by TEM. The white arrow in fraction 7 indicates the presence of protein contaminants, more frequently found in the latter SEC fractions. The scale bar displayed corresponds to 100 nm. (**B2**) Representative results of the particle size (blue bars; left axis) analyzed by TEM, and the amount of total protein quantified (yellow line; right axis) in each SEC fraction. Results are the mean ± standard error (SE) of a minimum of 200 EVs analyzed from each SEC fraction isolated from a PB sample (with the exception of fractions 3 and 4, in which only 6 and 18 EVs were analyzed, respectively). (**C**) EV markers and protein contaminants in SEC fractions 3 to 10 analyzed by WB. The blot displays representative results from markers found in PB (**C1**) and BM (**C2**) samples. The molecular weight (MW) of the bands is shown on the right side of each blot in kDa. Fractions 3 to 6 were ultrafiltrated separately. NTA: Nanoparticle Tracking Analysis; MM: Multiple Myeloma; PB: peripheral blood; BM: bone marrow; TEM: Transmission Electron Microscopy; WB: Western Blot.

Our results show the possibility of isolating EVs from the BM and PB of patients with MM. Choosing the optimal strategy to isolate EVs is a critical step in achieving enough of a yield of EVs with minimal contamination by non-vesicular proteins/particles in order to assure their accurate study [34,61]. SEC was previously described to be efficient in isolating EVs from plasma and separating them from some of the protein contaminants [62,63]. As seen in Figure 1(A2,A4,B1,B2), NTA and TEM analysis showed that the EVs of higher sizes are separated in the earlier fractions, while the EVs of smaller sizes are separated in later fractions. Particles between 50 and 1000 nm are mostly obtained on fractions 3 to 6 (Figure 1(A2,A4)). TEM analysis confirmed the NTA results, indicating that particle size decreases while the number of particles increases from fractions 3 to 7 (Figure 1A,B). The mean particle range determined by TEM was approximately between 80 and 40 nm for fractions 3 to 6, respectively (Figure 1B). Irrespective of the type of sample analyzed (PB or BM), the protein concentration rises considerably from fraction 7 onwards (Figure 1A,B), where the main protein contaminants such as albumin and apolipoprotein B are found in both PB and BM samples (Figure 1(C1,C2)). This is in agreement with other studies showing that SEC is an efficient method for isolating EVs from plasma samples and that from fractions 8 to 10 there is an increased level of contaminants [64,65,66,67,68]. TEM also showed the morphological heterogeneity of the isolated particles, with particles from fractions 3 to 6 being mostly round or with an irregular surface, as has been reported for EVs [57,69], contrasting with a more uniformed and spherical morphology found on particles isolated from fraction 7 onwards, as expected for apolipoproteins [64].

Finally, the presence of several EV markers (CD63, Mitofilin, CD9, CD81, Syntenin-1, TSG101, and Annexin XI) was found by WB in fractions 4 to 10 (Figure 1(C1,C2)). Most interestingly, when comparing the presence of EV markers between PB- and BM-isolated EVs, differences were found regarding Annexin XI, CD81, CD9, and CD63. Indeed, the PB EV marker found between fractions 3 and 6 was mostly CD9. However, in EVs isolated between fractions 3 and 6 from BM samples, the EV markers mostly found were Annexin XI and CD63. To our knowledge, this interesting finding had not been previously reported and suggests that even though the size of the EVs isolated (from fractions 3 to 6) is similar between PB and BM samples, those EVs have a different abundance of typical EV markers which therefore may correspond to distinct EV subsets. This difference may not reflect the protein content of the cells of origin of the isolated EVs, since selective packaging of proteins into EVs has been described [70,71,72]. This further enhances the relevance of comparing the cargo of MM-associated proteins in the EVs isolated from PB and BM samples.

The presence of EV markers in fraction 10, mainly Actinin4 and CD9 in PB EVs and CD81, Actinin4, and CD63 in BM EVs, might be due to the presence of these proteins in a free form, as EVs are not expected to be obtained from fractions 8 to 10, as demonstrated by the NTA and TEM results.

### 2.2. EVs Were Isolated from Diagnosis and Remission PB and BM Samples of MM Patients

**Figure 2:**
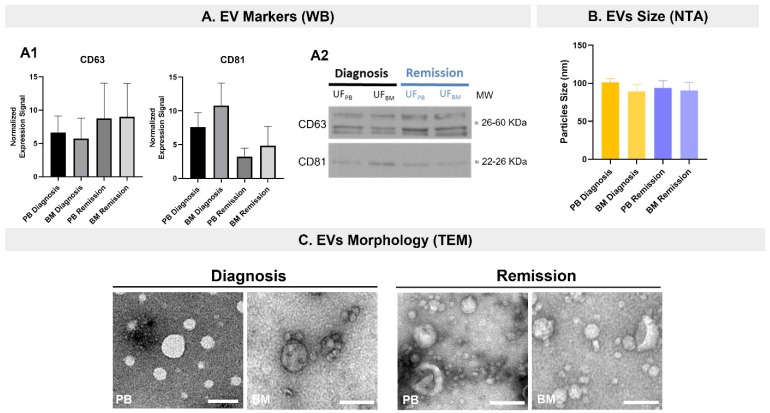
Characterization of the EVs isolated by Size Exclusion Chromatography (SEC) and concentrated by ultrafiltration (UF) from diagnostic and remission samples of peripheral blood (PB) and bone marrow (BM). (**A**) EV markers found by WB in the diagnostic and remission PB and BM EV samples. (**A1**) Quantification of CD63 and CD81 signals in the ultrafiltrated (UF) samples, normalized for the total protein of the lane (Ponceau staining). Results are the mean ± standard error (SE) from three MM patients. (**A2**) Representative blots of CD63 and CD81. (**B**) Average size of the isolated EVs. Results are the mean ± standard error (SE) from three MM patients. (**C**) Representative images of the isolated EVs. The scale bar displayed corresponds to 100 nm. PB: peripheral blood; BM: bone marrow; WB: Western Blot; UF: ultrafiltrated pool of SEC fractions 3 to 6; NTA: Nanoparticle Tracking Analysis; MM: Multiple Myeloma; TEM: Transmission Electron Microscopy.

It was possible to confirm the presence of EVs in PB and BM samples from MM patients obtained at either diagnosis or complete remission. The presence of the EV markers CD63 and CD81 was found in all samples (Figure 2A) without statistically significant differences, even though some differences in the levels of these markers were found at diagnosis and remission. Indeed, the levels of CD63 were lower at diagnosis than at the remission stage, while the levels of CD81 were higher at diagnosis than at the remission stage. This trend was observed both in the PB and BM samples. In agreement with what had been previously observed (Figure 1C), the EVs isolated from BM samples had higher levels of CD81 than the EVs isolated from PB samples, reflecting a difference in the protein cargo of EVs from both origins.

NTA results showed consistency regarding the EV sizes isolated from all samples (PB and BM at diagnosis and remission), which presented mean EV sizes between approximately 40 and 100 nm (Figure 2B), without statistically significant differences. This was confirmed by TEM (Figure 2C).

#### 2.2.1. EVs Isolated from Diagnostic PB and BM Samples Present MM Markers in Their Cargo

**Figure 3:**
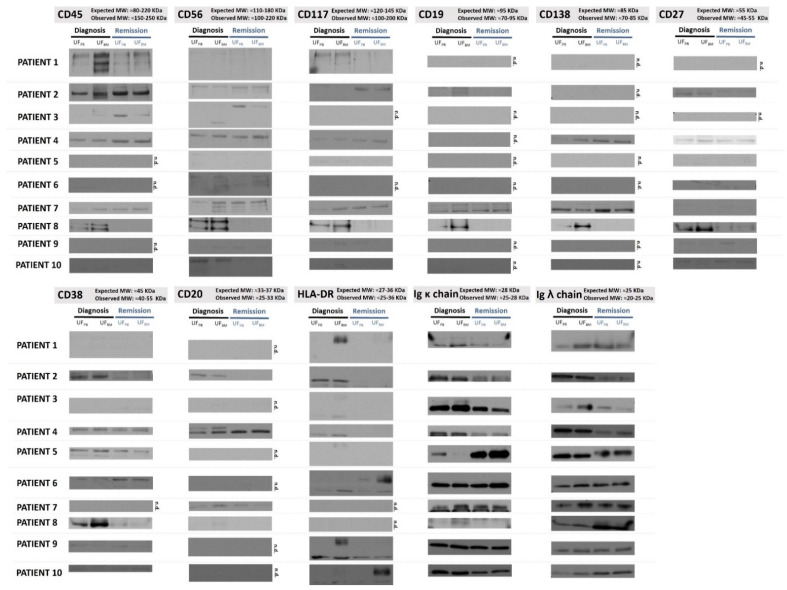
Detection of Multiple Myeloma (MM) and Minimal Residual Disease (MRD) Markers in EVs from diagnosis and remission samples from the peripheral blood (PB) and bone marrow (BM) of MM patients. Different markers of MM and MRD were analyzed by WB in the ultrafiltrated pool from SEC fractions 3 to 6 (UF) isolated from PB and BM samples collected at both diagnosis (first two lanes) and remission (final two lanes) from 10 MM patients (n.d.: signal not detected). The molecular weight (MW) of the bands is shown in kDa on the right side of the blots. MM: Multiple Myeloma; MRD: Minimal Residual Disease; PB: peripheral blood; BM: bone marrow; SEC: Size Exclusion Chromatography; WB: Western Blot; UF: ultrafiltrated pool from SEC fractions 3 to 6.

Different immunophenotypic markers of MM were analyzed by WB in the ultrafiltrated pools from SEC fractions 3 and 6 for both PB and BM patient samples at diagnosis and remission status. The MM EV markers analyzed consisted of the well-known MM markers related to specific PC lineages, such as CD38 and CD138, and others used by the Euroflow Consortium for the diagnosis and response evaluation to treatment by flow cytometry: CD45, CD56, CD19, CD81, CD117, CD27, and cytoplasmic immunoglobulin kappa and lambda light chains [73]. Results are presented in Figure 3, with the first two lanes of each blot presenting results from diagnostic samples (from PB and BM).

Levels of CD38 and CD138 were analyzed in ten patients. A higher number of CD38 positive samples were observed compared to CD138 and co-presentation of CD38/CD138. This co-presentation was particularly evident in patients 4 and 8. However, other patients, such as patient 1 and 3, did not present CD38+ in EVs at diagnosis. Recently, it was described that low levels of CD38 were found in PCs of patients with EMD, conferring poor response to anti-CD38 monoclonal antibodies. Interestingly, our patients without CD38 in EVs at diagnosis, and CD38-negative by flow cytometry (Table 1), did present EMD with low medullary plasmacytosis [74]. MM patients without CD38 are also reported to have worse prognosis while being refractory to treatment [75].

Both CD38 and CD138, as specific lineage markers, were described to be the best markers to use when identifying the source EVs derived from monoclonal PCs [24,57]. Nonetheless, it is necessary to carefully interpret results from CD38+ EVs and CD138+ EVs once they can derive from other cells rather than PCs. Indeed, CD38 is also detectable in low levels in other lymphoid cells (NK, B, and activated T cells) and myeloid cells (monocyte), while CD138 can be expressed in low levels by epithelial cells [76,77]. When possible, co-presentation of CD38/CD138 should be used to identify MM EVs [57]. In a recent study, these double positives showed that their concentration was on average two-fold greater in MM patients when compared to healthy controls, whereas single CD38+ or CD138+ positivity in EVs was lower (less than one-fold) when compared to healthy controls [57].

Expression of CD45 was found in EVs from six patients (Figure 3), and in half of them CD45 was more expressed in BM than in PB (patients 1, 2, and 8). The impact of CD45 positivity in MM at diagnosis is controversial in terms of prognostic value, despite being associated with higher proliferative cell rates [78]. It was difficult to establish a pattern between proliferation and the level of CD45 EVs in our patients.

Restriction for kappa (κ) or lambda (λ) light chains is usually used as a surrogate for clonality in MM, with abnormal ratios suggesting the presence of such a restriction [73]. Importantly, there were different levels of kappa or lambda light chains in the samples analyzed (Figure 3), with predominance of one of the light chains corresponding to the PC isotype defined by serum immunofixation (Table 1).

Other markers were reported to be increased in MM EVs, although they are not specific to MM and could be used as aberrant markers. For example, increased levels of the transmembrane glycoprotein CD147 were found in MM-derived EVs and appeared to be related to tumor cell growth and MM progression [79,80], while the adhesion molecule CD44 was increased in MM-derived EVs from patients treated with steroids and lenalidomide [81,82].

#### 2.2.2. MM Markers Can Be Found in EVs from Both BM and PB Samples

When comparing the presence of MM markers between paired PB and BM samples, it was possible to verify that, with some minor exceptions, most of the markers present in the EVs isolated from BM were also present in EVs isolated from PB (Figure 3). However, differences were found in the levels of those markers when comparing paired PB and BM samples for CD45 (patients 1, 2, 3, 7, and 8), CD56 (patient 3, 7, 8, and 10), CD19 (patient 2 and 8), CD138 (patients 4, 7, and 8), CD27 (patient 8), CD38 (patient 8 and 9), HLA-DR (patients 1, 3, 5, 6, and 10), immunoglobulin kappa (Ig κ) (patients 1, 5, 7, and 8), and immunoglobulin lambda (Ig λ) (patients 1, 3, 7, and 10). The observed differences were found mostly in the diagnostic samples. In most cases, the differences found at diagnosis consisted of higher levels in BM than in PB samples. However, and surprisingly, in the remission samples the levels of the markers were very similar between PB and BM paired samples. More similar results between PB and BM were only obtained for CD117 and CD20 in all analyzed patients.

Even though this WB analysis is not a quantitative analysis, and the analysis of results is limited by the lack of a proper EV protein loading control, this suggests that the cargo of immunophenotypic markers on EVs from BM and PB paired samples is not the same. Therefore, the search for these biomarkers in liquid biopsies (blood samples, PB) from diagnostic samples may not provide similar results to the ones obtained from BM aspirates. Nonetheless, similar levels of immunophenotypic markers are present in EVs from BM and PB at the remission stage, suggesting that the follow up of MM patients by analysis of EVs in liquid biopsies (PB samples) may be representative of the disease markers in the BM. This finding is of major relevance regarding the potential use of EVs as biomarkers in liquid biopsies for MRD monitoring.

Detection of MRD in PB mirroring BM and/or EMD would bring obvious advantages to patients and clinicians, allowing frequent and minimally invasive real-time monitoring of the disease [83,84,85,86]. In fact, the use of a liquid biopsy has been proposed as an alternative approach to monitoring MRD in MM and mainly uses circulating tumor DNA (ctDNA) [83,87,88], circulating tumor cells (CTCs) [89,90,91], serum monoclonal immunoglobulins [14], and, recently, EVs [44,92]. EVs are easy to assess and considered as possible minimally invasive biomarkers for cancer because of: (i) their capacity to carry important and relevant cargo that reflects the cell of origin; (ii) their ability to protect their cargo from external degradation; and (iii) their considerable longevity and stability in circulation [93]. These are major advantages compared to other circulating cell-free molecules such as proteins and microRNAs that are susceptible to degradation and have a short half-life [85,94]. Compared to CTCs, EV assessment requires a smaller sample volume to guarantee the likelihood of detection since they are easily found in PB [85]. The ctDNA also requires large volumes of plasma to be analyzed, having unpredictable half-lives compared to EVs [95]. Additionally, only 0.1–10% of the total circulating cell-free DNA (cfDNA) consists of ctDNA, with non-tumoral cfDNA hampering the use of ctDNA as a biomarker [85,96].

#### 2.2.3. EVs from Diagnosis and Remission PB and BM Patient Samples Present Different Levels of MM Markers

In all the ten patients included in the analysis, there was a lack of consistency regarding the levels of most MM markers found in the cargo of EVs isolated from paired diagnosis or remission samples (Figure 3). Nonetheless, in some patients, clear changes were found according to the stage of the disease in terms of the levels of some markers, such as CD38, CD45, CD56, and HLA-DR. 

Regarding the comparison of MRD status determined by flow cytometry and the EV MM markers found at remission, it is difficult to establish a definite correlation, even though some patterns can be found (Table 1). Indeed, in some patients, the disappearance at remission of specific markers in the EVs could support their possible use as MRD markers (Table 1). For example, regarding CD38 levels, results (Figure 3) show that there is a reduction in CD38 levels for patients 2 and 8 at remission when compared to diagnosis, both in PB- and BM-isolated EVs, possibly reflecting a change in the disease burden. Both patients presented a high-risk disease with International Staging System 3 (ISS 3) and amp1q21 in Fluorescence in situ hybridization (FISH) analysis (with bad prognosis) and also had a CR after an autologous stem cell transplant (ASCT) which achieved MRD negativity when assessed by flow cytometry (Table 1). It was previously reported that in EVs derived from MM-PCs expressing CD38, their quantity was positively correlated to the clinical staging system, in which the number of CD38+ EVs was significantly higher in patients with an ISS of 3, echoing aggressive disease, when compared to those with an ISS of 2 or ISS of 1 [46]. 

Interestingly, patient 8 also presented a disappearance of CD138 in EVs isolated from remission (in both PB and BM samples, Figure 3). In another study, positive CD138 EVs were associated with disease stage and therapeutic response, with CD138+ circulating EVs increasing gradually in patients with relapsed MM, reflecting the disease burden and resistance to treatment [97]. Another study evaluated CD138 as a possible marker in the PB to assess response and showed that the levels of CD138+ EVs were higher in newly diagnosed MM patients when compared to patients in remission or healthy donors, thus providing support for the use of EVs as a tool for monitoring MM in PB [98]. This suggested the possibility of using CD138+ EVs as a prognostic tool and surrogate of treatment response [97,98]. In fact, a specific resistance signature of CD138+/P-glycoprotein+(P-gp+)/ CD34+ EVs was found to be significantly elevated in the plasma of patients with aggressive disease and persistent residual disease, supporting its possible role as an MRD marker in EVs [99].

Strikingly, patient 8 presented a decrease in all analyzed MM markers in the remission sample, together with a decrease in kappa but an increase in lambda chains (Figure 3). The pattern observed may represent a CR to ASCT with recovery of polyclonality, restoration of the kappa/lambda ratio, and no residual disease detected in the EV cargo. This patient after ASCT reached a CR with normalization of the ratio of free light chains and negative residual disease as assessed by flow cytometry.

Interestingly, CD56 increased in remission for patient 3, while it was reduced or disappeared for patients 8 and 10 (Figure 3). The expression levels of CD56 in MM are reported to be up to 80% but its impact is controversial, being mainly associated with EMD and worse prognosis [100,101]. Patient 3 had no EMD, but had a revised-ISS of 3 and t(4;14) (Table 1), which is associated with severe prognosis; at day 100 after ASCT this patient did not achieve negative residual disease [102]. 

Regarding CD45, its levels decreased on the remission EV samples from patient 1 and disappeared on the remission EV samples from patient 8. However, its levels increased in the remission samples of patients 2, 3, and 4 (Figure 3). It is accepted that, during normal PC development and differentiation, CD45 levels progressively reduce, but its role in MM is controversial. Its persistence appears to reflect more immature PCs and an aggressive phenotype [78]. As with patients 2 and 3 with high ISS or revised-ISS (R-ISS) stages and high-risk features in cytogenetics harboring bad prognosis, patient 4 also had bad prognosis with an R-ISS of 3 and double the amount of high-risk features in the cytogenetics analysis, specifically t(4;14) and del17p (Table 1). The increased levels of CD45 in MM EVs may reflect drug resistance and may be implicated in a compromised depth of response, with both patients 3 and 4 being positive in the flow cytometry MRD assessment at day 100 after ASCT (Table 1).

HLA-DR also presented different levels at diagnosis and remission, being reduced at remission in most patients but intensively increased in the BM EVs from patients 6 and 10 (Figure 3). The HLA-DR antigen most frequently appears on the cytomembrane of macrophages and B lymphocytes and may aid the host immune system in identifying and attacking tumor cells. Its relation to MM is less explored but its persistence is associated with shorter survival [103]. Nonetheless, it was not possible to establish a pattern between these bad prognoses and our patients. Patient 6 had no cytogenetic high-risk features while patient 10 had an R-ISS of 3 and t(4;14) (Table 1).

### 2.3. EVs from Healthy Controls Do Not Present Most of the MM Markers

**Figure 4:**
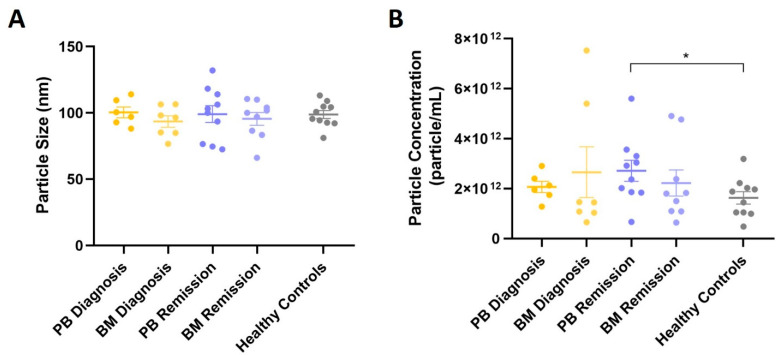
Comparison of the size and concentration of the EVs isolated by Size Exclusion Chromatography (SEC) and concentrated by ultrafiltration (UF) from Multiple Myeloma (MM) patients and healthy controls. (**A**) Average size of the isolated EVs from PB and BM samples of both diagnosis and remission MM patients and from healthy controls. Results are the mean ± standard error (SE) of the particle size obtained by NTA from at least six samples. (**B**) Particle concentration of the isolated EVs from PB and BM samples from both diagnosis and remission MM patients and from healthy controls. Results are the mean ± standard error (SE) of particle concentration obtained by NTA from at least six samples. * *p* < 0.05. PB: peripheral blood; BM: bone marrow; SEC: Size Exclusion Chromatography; UF: ultrafiltrated pool of SEC fractions 3 to 6; NTA: Nanoparticle Tracking Analysis; MM: Multiple Myeloma.

**Figure 5:**
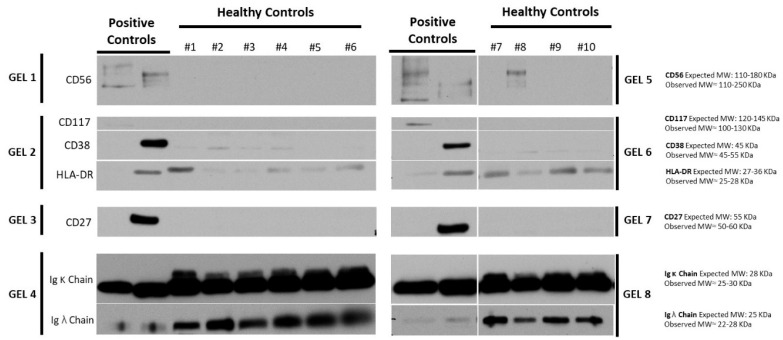
Analysis of Multiple Myeloma (MM) and Minimal Residual Disease (MRD) markers in EVs from the peripheral blood (PB) of healthy donors. Different markers of MM and MRD were analyzed by WB in the ultrafiltrated pool of SEC fractions 3 to 6 (UF) from the PB of 10 healthy donors. The UF from two MM patients was used as positive controls (left side of the blots). The molecular weight (MW) of the bands is shown in kDa on the right side of the blots. MM: Multiple Myeloma; MRD: Minimal Residual Disease; PB: peripheral blood; BM: bone marrow; SEC: Size Exclusion Chromatography; WB: Western Blot; UF: ultrafiltrated pool of SEC fractions 3 to 6.

The same markers of MM and MRD were analyzed in the EVs isolated from the PB of ten healthy controls by WB of the ultrafiltrated pool of SEC fractions 3 to 6 (UF). Results are presented in Figure 4 and Figure 5. EVs concentration was lower in the PB of healthy controls than in the PB or BM of patients with MM (even though only the comparison between PB of healthy controls and PB of patients at remission was considered to be statistically significant) (Figure 4B), while the average size of the isolated EVs was very similar (Figure 4A). Overall, the levels of EVs in cancer, including hematological malignancies, are reported to be considerably higher than in healthy controls [46], highlighting their potential as cancer biomarkers [43].

As expected, IgG kappa and lambda were detected in EVs isolated from healthy donors (Figure 5). However, HLA-DR is also detected in healthy controls, indicating that this is not a suitable biomarker to be used for MM diagnosis or monitoring of MRD. However, some MM markers (CD56, CD117, CD27) were not identified in healthy controls, with the exception of control 8 for CD56. In addition, CD38 was barely detected in healthy controls (Figure 5). This agrees with other studies, which found CD38+ EVs in MM serum but not in healthy controls [46,98]. A pivotal study previously demonstrated that serum samples from patients with PC diseases contained higher levels of Hsp70, Annexin IV, and c-Src-positive EVs derived from monoclonal PCs than healthy controls [104]. Additionally, the quantity of serum EVs expressing CD38 [46,98] or CD138 [97,99,105] was found to be significantly elevated in MM patients compared to healthy controls and related to disease severity [46], stage, and response/resistance to treatment [97,99,105].

The absence of MM markers in EVs from healthy controls further suggests that markers such as CD38, CD56, CD117, and CD27 are suitable for use as biomarkers of MM or in response monitoring of MRD.

## 3. Material and Methods

### 3.1. Patients and Samples

Patient peripheral blood (PB) and bone marrow (BM) samples were collected from Multiple Myeloma (MM) patients at diagnosis and at remission on the 100th day after autologous stem cell transplant (ASCT) at the Clinical Hematology Department from Centro Hospitalar Universitário São João (CHUSJ, Porto, Portugal). All patients were newly diagnosed with MM and treated upfront with the triplet combination of bortezomib, thalidomide, and dexamethasone. Treatment response on the 100th day after ASCT was assessed according to the International Multiple Myeloma Group recommendations [13,14,24,106].

### 3.2. Minimal Residual Disease (MRD) Determination by Flow Cytometry

All Minimal Residual Disease (MRD) assays were performed on the BM aspirates within 24 h of collection according to the Eight Color EuroFlow panel for MM, combining surface antigens for the identification of phenotypically abnormal clonal plasma cells (PCs)—CD38, CD138, CD45, CD19, CD56, CD27, CD81, and CD117—and cytoplasmic lambda and/or kappa light chains to confirm clonality. MRD was considered negative with a cut-off level of less than 20 clonal PCs in 2 million nucleated cells (minimal sensitivity of 10^−5^) and the limit of detection (LOD) in each assay was determined according to the formula (20/nucleated cells) × 100. Any patient was considered MRD negative if there was an absence of clonal PCs or if they were present below the LOD achieved in the corresponding sample [24,107,108].

### 3.3. Platelet-Poor Plasma (PPP) Isolation

Samples were collected in tubes buffered with 3.8% of sodium citrate (Vacutest^®^ KIMA, Arzergrande, Italy). The PB and BM samples were transferred to centrifuge tubes using an aseptic technique, and an equal amount of phosphate-buffered saline 1× (PBS 1×) was added to each. The mix was gently transferred to tubes containing Histopaque-1077 (Sigma-Aldrich, Steinheim, Germany) and centrifuged for 30 min at room temperature (RT) at 400× *g*. The Platelet-Rich Plasma (PRP) was centrifuged (at 2500× *g*, at RT, for 15 min), resulting in Platelet-Poor Plasma (PPP). The PPP was divided into aliquots and immediately stored at −80 °C until further use. 

### 3.4. Extracellular Vesicles (EVs) Isolation from Platelet-Poor Plasma (PPP)

#### 3.4.1. Size Exclusion Chromatography (SEC)

The isolation of Extracellular Vesicles (EVs) was performed using a previously described method with some alterations [62]. After being washed with filtered 0.32% (*w/v*) trisodium citrate dihydrate (Merck Life Science, Darmstadt, Germany) in PBS (PBS-citrate, pH 7.4), Sepharose cross-linked 2B (CL2B300, Merck Life Science, Darmstadt, Germany) was placed in a 10 mL syringe (10 mL SOFT-JECT^®^, Henke Sass Wolf, Tuttlingen, Germany) with its tip filled with a piece of nylon stocking (20 denier). When the compacted Sepharose reached the 11 mL mark, a 3 MM paper filter (3 MM CHR; Cytiva, Marlborough, MA, USA) was added to the top. The column was kept in 20% ethanol and stored at 4 °C until further use.

For EVs isolation, the 20% ethanol solution was removed by loading PBS-citrate into the SEC column. The PPP (1 mL) was loaded with the continuous addition of PBS-citrate. Ten sequential 1 mL fractions were collected. Fractions were either used immediately for EVs characterization or stored at −80 °C until further use. 

#### 3.4.2. Ultrafiltration (UF)

To concentrate the EVs, around 4 mL from pools of EV-rich and protein-low fractions (i.e., SEC fractions 3 to 6) was filtered with a 100 kDa cut-off membrane (Amicon^®^ Ultra-4 Centrifugal Filters Ultracel^®^—100 K, Milipore, Merck Life Science, Darmstadt, Germany) and centrifuged at 4 °C at 3200× *g*. The ultrafiltrated (UF) sample was collected, quantified (as described below), and stored at −80 °C until downstream analysis.

### 3.5. Protein Quantification

The protein amount of each SEC fraction (either from the EV cargo or protein contaminants) was determined using the Lowry protein assay (DC™ Protein Assay kit, Bio-Rad, Hercules, CA, USA), according to the manufacturer’s instructions. The absorbance was analyzed in a microplate reader (Synergy™ Mx, BioTek Instruments Inc., Winooski, VT, USA) with a 488 nm excitation wavelength and read at the 655 nm emission.

### 3.6. EVs Characterization

#### 3.6.1. Nanoparticle Tracking Analysis (NTA)

Particle concentration and size distribution were obtained by Nanoparticle Tracking Analysis (NTA). The SEC fractions and the UF sample were pre-diluted (1:10 to 1:10,000) in 0.22 µm of filtered PBS-citrate (to reach the optimal concentration read-out range of 10^7^ to 10^9^ particles/mL). The samples were loaded at a constant rate in a NanoSight NS300 (Malvern Instruments Ltd., Malvern, UK) with a 1 mL syringe (Omnifix^®^ 100 Solo, B|BRAUM, Melsungen, Germany) using a Nanosight syringe pump (Malvern Instruments Ltd., Malvern, UK) at RT. Three separate 30 s videos were recorded with the following specifications: camera type, sCMOS; laser type, Blue488; camera level, 15/16; slider shutter, 1206/1300; slider gain, 366/512; FPS, 25.0; temperature, 21.1–25.5 °C; viscosity, 0.878–0.974 cP; syringe pump speed, 40. Particles were detected by video analysis using NanoSight NTA Software (NTA version 3.2, Dev Build 3.2.16) with the following settings: detection threshold, 5; blur size, auto; max jump distance, auto (8.3–16.2 pixels). The mean, mode, and median vesicle size (nm) and estimation of the particle concentration (particles/mL) were determined. The data obtained by NTA were then analyzed using GraphPad (Prism 8).

#### 3.6.2. Transmission Electron Microscopy (TEM)

EVs size, morphology, and integrity were visualized by Transmission Electron Microscopy (TEM) using negative staining. Each sample was resuspended in PBS-citrate or in a 1:2 solution of PBS-citrate and HEPES 20 mM (Merck Life Science, Darmstadt, Germany) +4% (*w/v*) sucrose (Merck Life Science, Darmstadt, Germany), mounted in Formvar-carbon-coated electron microscopy grids (Electron Microscopy Sciences, Hatfield, PA, USA) for 2 min in the dark at RT, and dried with a filter paper. TEM grids were then counterstained with 5% uranyl acetate and visualized under the transmission electron microscope (Jeol JEM 1400, JEOL, Tokyo, Japan) with an acceleration voltage of 80 kV. The data were obtained by the Histology and Electron Microscopy Service, i3S, Porto, Portugal. Representative TEM photographs were acquired and the EV size for each SEC fraction was measured by image analysis using ImageJ software.

#### 3.6.3. Western Blot (WB)

The SEC fractions and the UF sample were denatured in loading buffer (Tris-HCl 1 M pH 6.8, 10% SDS, 85% glycerol, β-mercaptoethanol, 1% bromophenol blue) and boiled at 95 °C for 5 min. A total of 15 μg of protein from each fraction was separated using SDS-PAGE (Mini-PROTEAN^®^ Tetra Vertical Electrophoresis Cell, Bio-Rad, Hercules, CA, USA) and transferred to a nitrocellulose membrane (GE Healthcare Life Science, Chalfont St Giles, UK) using a Mini Trans-Blot^®^ cell system (Bio-Rad, Hercules, CA, USA). After the transfer, the proteins were stained with Ponceau S Solution (PanReac AppliChem, Barcelona, Spain) and images were acquired with Chemidoc XRS+ System equipment (Bio-Rad, Hercules, CA, USA). The membranes were blocked for at least 2 h at RT in a blocking solution consisting of 5% (*w/v*) non-fat dry milk (Molico, Nestlé, Vevey, Switzerland) in TBS-T (Tris-buffered saline solution pH 7.4 with 0.1% Tween-20, [Promega Corporation, Madison, WI, USA]). After blocking, membranes were incubated with primary antibodies (Santa Cruz Biotechnology, Dallas, TX, USA; GeneTex, Irvine, CA, USA) diluted in the blocking solution in accordance with the manufacturer’s instructions (Supplementary Table S1) and stirred for 90 min at RT. After being washed in TBS-T, membranes were incubated with the secondary antibodies (GE Healthcare Life Science, Chalfont St Giles, UK; Santa Cruz Biotechnology, Dallas, TX, USA) (Supplementary Table S2) for 1 h with agitation at RT. The signal of the membranes was then detected using the enhanced chemiluminescence (ECL) Western Blotting Detection Reagent (GE Healthcare Life Science, Chalfont St Giles, UK), Amersham Hyperfilm ECL (GE Healthcare Life Science, Chalfont St Giles, UK), and a Fuji Medical Film Processor (FPM-100A Model, Fuji Photo, Tokyo, Japan). The molecular weight of protein bands was estimated by comparison with an established protein marker (PageRuler™ Plus Prestained Protein Ladder, 10 to 250 kDa, ThermoFisher Scientific, Waltham, MA, USA). Band quantification was carried out using Image Lab^TM^ Software version 6.0.1 (Bio-Rad, Hercules, CA, USA). Signal quantification of the bands was normalized to the total protein of the lane, which was obtained by Ponceau staining.

### 3.7. Multiple Myeloma (MM) Marker Analysis by Western Blot (WB)

A total of 15 μg of the UF samples from the PB and BM patient samples and from the PB of healthy donors was denatured and separated by SDS-PAGE followed by WB using the same protocol and systems as described above. Antibodies (GE Healthcare Life Science, Chalfont St Giles, UK; Santa Cruz Biotechnology, Dallas, TX, USA; Abcam, Cambridge, MA, USA) for MM-specific markers (such as CD38 and CD138) were used, as well as other antibodies relative to the antigens used in the EuroFlow panel, such as CD19, CD27, CD45, CD81, CD56, CD117, and cytoplasmic lambda and/or kappa light chains [13,24] (Supplementary Tables S2–S4, and Figure S1).

### 3.8. Statistical Analysis

The statistical analysis was performed using the two-tailed unpaired *t*-test which was obtained in GraphPad Prism 8.0 software. Statistical significance was considered whenever *p* < 0.05.

## 4. Conclusions

In the present study, we demonstrated the possibility of isolating EVs from the BM and PB of MM patients at distinct disease stages (diagnosis and remission) and compared the MRD-associated markers between paired BM and PB EVs at diagnosis and remission stages. Firstly, our results demonstrated that EVs from BM and PB samples, both from diagnosis and remission, were successfully isolated using the SEC method, concentrated by UF, and characterized by NTA, TEM, and WB in terms of size distribution, morphology, and the presence of well-known EV protein markers. Secondly, EVs isolated from PB or BM at diagnosis present specific MM markers, such as CD38 and CD138, confirming the potential of EVs as MM biomarkers. Thirdly, by analyzing alterations in the levels of MM markers in EVs isolated from paired diagnosis and remission samples, it was possible to verify differences between those markers, enhancing the possibility of using EVs to monitor MRD. Fourthly, even though in the diagnostic samples the levels of MM markers were different in several PB and BM samples, in the remission samples those levels were very similar, thus suggesting that EVs have the potential to monitor MRD in liquid biopsies from remission samples. Finally, the much lower levels of MM markers in the EVs isolated from the PB of healthy controls supports the possibility of using those markers as specific MM biomarkers to non-invasively monitor MRD. 

Nonetheless, some caveats are exposed in our study. It was not powered to provide definite clinical advice; thus, further studies should be attempted to extend this study to a larger sample of MM patients and integrate this approach with NGS or NGF at different disease timepoints. Moreover, even if the SEC method proved to be efficient in isolating EVs and the protocols to characterize those EVs were well described, overall, the methodology is time consuming and needs to be further optimized to become reproducible and easier to use in clinical practice. Furthermore, there is no standardization in the interpretation of results, which relies on sample-by-sample analysis by experienced operators.

Overall, this study suggests the possibility of isolating EVs from PB samples of MM remission patients for use in the monitoring of MRD. Our results warrant a prospective and larger study with more samples from MM patients to confirm and reproduce the herein presented data, to optimize the methodology in order for it to become easier to use in clinical practice, and to determine its sensitivity regarding MRD detection so that it can possibly assume its role as a method for analyzing non-invasive liquid biopsies of MM.

## Figures and Tables

**Table 1:** Comparison of Multiple Myeloma (MM) patients’ characteristics at diagnosis and at day 100 of evaluation regarding response criteria, measurable residual disease (MRD) by flow cytometry, and Extracellular Vesicle (EV) markers both in peripheral blood (PB) and bone marrow (BM).

Patient	MM Isotype	ISS	FISH	EMD	% of PCs at Diagnosis		MM Markers by WB at EVs PB/BM at Diagnosis				IMWG Response Criteria at D100	MRD at D100 by Flow Cytometry	% of PCs at D100		MM Markers by WB at EVs PB/BM at D100			
					PB	BM	CD38	CD138	K	L	BM	BM	PB	BM	CD38	CD138	K	L
01	IgG/K	2	no abnormality	Yes	≤0.05	≤10	wd/wd	nd	++/++	+/++	sCR	negative	0	≤0.05	wd/−	nd	+/+	++/+
02	IgG/K	3	amp1q21	No	0	≥40 and ≤50	++/++	nd	++/++	++/++	sCR	negative	0	≥0.05 and ≤0.1	+/+	nd	+/+	+/+
03	IgG/K	3	t(4;14)	Yes	≤0.05	≤10	−/−	nd	++/++	+/++	sCR	positive	≤0.01	≥0.1 and ≤0.5	wd/wd	nd	+/+	+/wd
04	IgA/L	3	t(4;14), del17p	No	≤0.05	≥10 and ≤20	++/++	+/++	++/++	++/++	VGPR	positive	≤0.01	≥0.1 and ≤0.5	++/++	++/++	+/+	+/+
05	IgG/L	2	no abnormality	No	≤0.002	≥10 and ≤20	++/++	nd	+/wd	++/++	sCR	positive	0	≤0.05	++/+	nd	++/++	++/++
06	L	3	no abnormality	No	≤0.002	≥20 and ≤30	+/+	nd	++/++	+/+	sCR	negative	0	≤0.05	++/++	nd	++/++	+/+
07	K	3	del17p	Yes	0	≤10	nd	++/+	+/++	+/++	CR	negative	0	≤0.05	nd	++/+	+/+	++/++
08	IgG/L	3	amp1q21	No	≤0.05	≥40 and ≤50	+/++	+/++	wd/+	+/+	sCR	negative	≤0.01	≤0.05	wd/wd	−/−	wd/wd	++/++
09	IgG/K	2	no abnormality	No	0	≥30 and ≤40	+/wd	nd	++/++	+/+	sCR	negative	0	≤0.05	+/wd	nd	++/++	+/+
10	K	3	t(4;14)	Yes	0	≥10 and ≤20	wd/−	nd	++/++	wd/+	CR	positive	0	≤0.05	wd/wd	nd	+/+	++/++

BM: bone marrow; CR: complete response; D100: day 100 after autologous stem cell transplant; EMD: extramedullary disease; EVs: Extracellular Vesicles; FISH: Fluorescence in situ hybridization; Ig: immunoglobulin; IMWG: International Myeloma Working Group; ISS: International Staging System; K: immunoglobulin kappa (κ) chain; L: immunoglobulin lambda (λ) chain; MM: Multiple Myeloma; MRD: measurable residual disease; nd: signal non-detectable in all samples in the same WB; PB: peripheral blood; PCs: plasma cells; sCR: stringent complete response; VGPR: very good partial response; WB: Western Blot; wd: signal weakly detected in WB; (−) negative in WB; (+) positive in WB; (++) intense positive in WB.

## Data Availability

The datasets generated and/or analyzed during the current study are available from the corresponding author on reasonable request, provided no ethical, legal, or privacy issues are raised.

## Supplementary Materials

The following supporting information can be downloaded at: https://www.mdpi.com/article/10.3390/ijms232213686/s1.

## Abbreviations

AS-PCR	allele-specific oligonucleotide polymerase chain reaction
ASCT	autologous stem cell transplant
BM	bone marrow
CAR-T cells	chimeric antigen receptor-T cells
CHUSJ	Centro Hospitalar Universitário São João
cfDNA	cell-free DNA
CTCs	circulating tumor cells
ctDNA	circulating tumor DNA
CR	complete response
D100	day 100 after ASCT
ECL	enhanced chemiluminescence
EMD	extramedullary disease
EVs	Extracellular Vesicles
FDG-PET/CT	18-fluoro-2-deoxyglucos positron emission tomography/computed tomography
FISH	Fluorescence in situ hybridization
FPM	Fuji Medical Film Processor
Ig	immunoglobulin
IMWG	International Myeloma Working Group
ISS	International Staging System
K	immunoglobulin kappa (κ) chain
L	immunoglobulin lambda (λ) chain
LOD	limit of detection
MFC	multiparameter flow cytometry
MM	Multiple Myeloma
MRD	measurable residual disease
MSCs	BM mesenchymal stem cells
MW	molecular weight
NGF	next-generation flow
NGS	next-generation sequencing
NTA	Nanoparticle Tracking Analysis
OS	overall survival
PB	peripheral blood
PBS	phosphate-buffered saline
PC	plasma cells
PFS	progression free-survival
PPP	Platelet-Poor Plasma
PRP	Platelet-Rich Plasma
R-ISS	revised-ISS
RT	room temperature
sCR	stringent complete response
SEC	Size Exclusion Chromatography
TBS-T	Tris-buffered saline solution with 0.1% Tween-20
TEM	Transmission Electron Microscopy
UF	ultrafiltration
UF	ultrafiltrated pool from SEC fractions 3 to 6
VEGF	vascular endothelial growth factor
VGPR	very good partial response
WB	Western Blot
