# Supplementary material for: Detection of Measurable Residual Disease Biomarkers in Extracellular Vesicles from Liquid Biopsies of Multiple Myeloma Patients—A Proof of Concept
Source: Int J Mol Sci. 2022 Nov 8;23(22):13686. doi: 10.3390/ijms232213686 (PMC9690807; doi:10.3390/ijms232213686)
Supplement: Supplementary file 1 [file ijms-23-13686-s001.zip › ijms-1978616-supplementary.pdf]

Supplementary Data

Detection of measurable residual disease biomarkers in extracellular vesicles from liquid biopsies of Multiple Myeloma patients – a proof of concept

Rui Bergantim [1,2,3,4], Sara Peixoto da Silva [1,2], Bárbara Polónia [1,2], Mélanie A. G. Barbosa [1,2], André Albergaria [1,5], Jorge Lima [1,5], Hugo R. Caires [1,2], José E. Guimarães [1,2,3,4,6,*] and M. Helena Vasconcelos [1,2,7,*]

[1] i3S—Instituto de Investigação e Inovação em Saúde, University of Porto, 4200-135 Porto, Portugal
[2] Cancer Drug Resistance Group, IPATIMUP—Institute of Molecular Pathology and Immunology of the University of Porto, 4200-135 Porto, Portugal
[3] Clinical Hematology, Hospital Center of São João, 4200-319 Porto, Portugal
[4] Clinical Hematology, FMUP—Faculty of Medicine of the University of Porto, 4200-319 Porto, Portugal
[5] Research Innovation Unit, Translational Research & Industry Partnerships Office, i3S—Instituto de Investigação e Inovação em Saúde, University of Porto, 4200-135 Porto, Portugal
[6] Instituto Universitário de Ciências da Saúde, Cooperativa de Ensino Superior Politécnico e Universitário IUCSESPU, 4585-116 Gandra-Paredes, Portugal
[7] Department of Biological Sciences, FFUP—Faculty of Pharmacy of the University of Porto, 4050-313 Porto, Portugal
* Correspondence: jeteguimaraes@gmail.com (J.E.G.); hvasconcelos@ipatimup.pt (M.H.V.); Tel.: +351-225-570-772 (J.E.G. & M.H.V.)

Supplementary tables

Supplementary Table S1. Analyzed EV markers and protein contaminants and their respective antibody dilutions.

Protein	Reference	Dilution factor	Expected Molecular Weight (KDa)
Actinin 4	GTX113116 (GeneTex)	1:500	105
Albumin	sc-271605 (Santa Cruz)	1:100	66
Alix	sc-49268 (Santa Cruz)	1:400	95
Annexin XI	sc-46686 (Santa Cruz)	1:100	50-56
ApoA-I	sc-376818 (Santa Cruz)	1:100	28
ApoB	sc-393636 (Santa Cruz)	1:100	512
CD63	sc-5275 (Santa Cruz)	1:200	26-60
CD81	sc-7637 (Santa Cruz)	1:200	22-26
CD9	sc-13118 (Santa Cruz)	1:200	24
Cytochrome C	sc-13560 (Santa Cruz)	1:200	15

HSP70	sc-66048 (Santa Cruz)	1:200	70
Mitofilin	sc-390707 (Santa Cruz)	1:100	88/90
Syntenin-1	sc-100336 (Santa Cruz)	1:200	33
TSG101	sc-136111 (Santa Cruz)	1:200	45

Supplementary Table S2: Secondary Antibodies and their respective dilution factors.

Antibody	Reference	Dilution Factor
Anti-mouse	NA931V (GE Healthcare)	1:2000
Anti-goat	sc-2354 (Santa Cruz)	1:2000
Anti-rabbit	NA934V (GE Healthcare)	1:2000

Supplementary Table S3: Analyzed Multiple Myeloma Markers and their respective antibody dilutions.

Protein	Reference	Dilution factor	Expected Molecular Weight (KDa)
CD56 (NCAM)	sc-7326 (Santa Cruz)	1:200	110-180
CD79B	Sc-18902 (Santa Cruz)	1:200	39
CD117 (c-kit)	sc-17806 (Santa Cruz)	1:100	120/145
CD138 (Syndecan-1)	sc-12765 (Santa Cruz)	1:200	85
CD38	sc-374650 (Santa Cruz)	1:100	45
HLA-DR	sc-53319 (Santa Cruz)	1:200	27/36
CD45	sc-1178 (Santa Cruz)	1:200	180-220
CD19	sc-373897 (Santa Cruz)	1:100	95
CD28	sc-70612 (Santa Cruz)	1:200	44
Ig λ chain [EPR5367-62]	ab124719 (abcam)	1:20000	25
Ig κ chain	sc-59265 (Santa Cruz)	1:200	28
CD44 (HCAM)	sc-9960 (Santa Cruz)	1:200	90-95
CD20	sc-393894 (Santa Cruz)	1:100	33-37
CD27	sc-25289 (Santa Cruz)	1:150	55

Supplementary Table S4: Expected and observed Molecular weights for the bands of the Multiple Myeloma and Minimal Residual Disease protein Markers, analyzed in both human samples and in cell lines of hematological malignancies. n.d. – not detected

Protein	Expected Weight (KDa)	Observed Molecular Weight (KDa)	
		Human Samples	Cell Lines
CD56	110-180	100-220	150-250+
CD45	80-220	150-250	200-250
CD117	120-145	100-200	n.d.
CD19	95	70-95	70-95
CD138	85	70-85	70-100
CD27	55	45-55	50
CD38	45	40-55	45
CD20	33-37	25-33	25
HLA-DR	27-36	25-36	25-37
Ig κ Chain	28	25-28	25
Ig λ Chain	25	20-25	20-25

Supplementary Figures

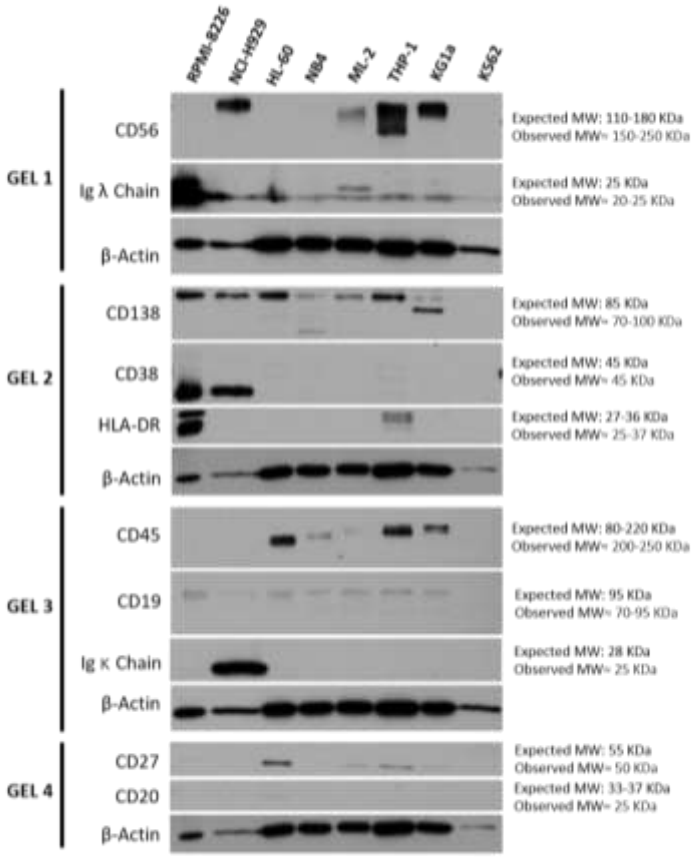

Supplementary Figure S1: Analysis of Multiple Myeloma (MM) and Minimal Residual Disease (MRD) protein Markers in cell lines of hematological malignancies. Different markers of MM and MRD were analyzed by WB in cell lysates of several cell lines of hematological malignancies. Actin staining was used as loading control. The molecular weight (MW) of the bands is shown in KDa on the right side of the blots.
